# Limited HIV Infection of Central Memory and Stem Cell Memory CD4+ T Cells Is Associated with Lack of Progression in Viremic Individuals

**DOI:** 10.1371/journal.ppat.1004345

**Published:** 2014-08-28

**Authors:** Nichole R. Klatt, Steven E. Bosinger, Melicent Peck, Laura E. Richert-Spuhler, Anke Heigele, Jillian P. Gile, Nirav Patel, Jessica Taaffe, Boris Julg, David Camerini, Carlo Torti, Jeffrey N. Martin, Steven G. Deeks, Elizabeth Sinclair, Frederick M. Hecht, Michael M. Lederman, Mirko Paiardini, Frank Kirchhoff, Jason M. Brenchley, Peter W. Hunt, Guido Silvestri

**Affiliations:** 1 Department of Pharmaceutics, Washington National Primate Research Center, University of Washington, Seattle, Washington, United States of America; 2 Laboratory of Molecular Microbiology, National Institute of Allergy and Infectious Disease, National Institutes of Health, Bethesda, Maryland, United States of America; 3 Yerkes Primate Research Center, Emory Vaccine Center and Department of Pathology, Emory University, Atlanta, Georgia, United States of America; 4 Department of Medicine, University of California San Francisco, San Francisco, California, United States of America; 5 Institute of Molecular Virology, Ulm University Medical Center, Ulm, Germany; 6 Ragon Institute of MGH, MIT and Harvard, Boston, Massachusetts, United States of America; 7 Institute for Immunology, University of California Irvine, Irvine, California, United States of America; 8 Institute of Infectious and Tropical Diseases, University of Brescia, Brescia, Italy; 9 Division of Infectious Diseases, Case Western Reserve University/University Hospitals Case Medical Center, Cleveland, Ohio, United States of America; Vaccine Research Center, National Institutes of Health, United States of America

## Abstract

A rare subset of HIV-infected individuals, designated viremic non-progressors (VNP), remain asymptomatic and maintain normal levels of CD4+ T-cells despite persistently high viremia. To identify mechanisms potentially responsible for the VNP phenotype, we compared VNPs (average >9 years of HIV infection) to HIV-infected individuals who have similar CD4+ T-cell counts and viral load, but who are likely to progress if left untreated (“putative progressors”, PP), thus avoiding the confounding effect of differences related to substantial CD4+ T cell depletion. We found that VNPs, compared to PPs, had preserved levels of CD4+ stem cell memory cells (T_SCM_ (*p*<0.0001), which was associated with decreased HIV infection of these cells in VNPs (r = −0.649, *p* = 0.019). In addition, VNPs had decreased HIV infection in CD4+ central memory (T_CM_) cells (*p* = 0.035), and the total number of T_CM_ cells was associated with increased proliferation of memory CD4+ T cells (r = 0.733, *p* = 0.01). Our results suggest that, in HIV-infected VNPs, decreased infection of CD4+ T_CM_ and T_SCM_, cells are involved in preservation of CD4+ T cell homeostasis and lack of disease progression despite high viremia.

## Author Summary

Here we assessed correlates of protection from disease progression in a rare subset of HIV-infected individuals, viremic non-progressors (VNP). These individuals have high viral load for several years. In contrast to the majority of infected individuals, however, these individuals do not progress to AIDS. Here we found this lack of progression was associated with selective preservation of two critical subsets of memory CD4+ T cells, central memory (T_CM_) and stem-cell memory (T_SCM_) cells. Compared to HIV-infected putative progressors, VNPs had higher proliferation of these indispensable subsets of memory cells. In addition, the long-lived CD4+ T_CM_ and T_SCM_ cells in VNPs had decreased HIV infection compared to the less critical effector memory CD4+ T cells, which indicates a possible mechanism by which VNPs maintain their CD4+ T cell pool after several years of infection, and remain free from AIDS progression.

## Introduction

In the majority of cases, HIV infection is characterized by high levels of viral replication, progressive loss of CD4+ T cells, and if left untreated, eventual progression to AIDS [1]. Predominant correlates of disease progression include HIV replication, loss of CD4+ T cells, high levels of pro-inflammatory cytokines, cell cycle dysregulation, signs of lymphocytic exhaustion, and up-regulation of type I interferon-stimulated genes (ISGs) [2], [3]. However, there is marked variability in the rate of HIV disease progression, largely due to host immune and genetic factors, as well as virus replication [4], [5]. Even an apparent relatively stable asymptomatic course of infection is observed in a small subset of patients, termed long-term non-progressors (LTNP) [1], [6]–[9]. The vast majority of LTNP maintain relatively high CD4+ T cell counts and low levels of immune activation by controlling viral replication to very low levels, often through vigorous CD8+ T cell mediated immune responses that occur in individuals with certain “protective” HLA class I alleles including HLA-B57 and HLA-B27 [7], [8], [10]. In particular, a striking feature in LTNPs which is associated with protection from disease progression is maintenance of long-lived CD4+ central memory T cells (T_CM_) which are essential for long-term immunological memory [11]–[13]. In addition, Descours et al. recently described that decreased HIV infection of T_CM_ cells in HLA-B57/B27 positive LTNPs was associated with HIV-specific CD8+ T cell responses, and is a mechanism of T_CM_ preservation and lack of disease progression [9].

More recently, a rare population of HIV infected individuals has been described who do not progress to AIDS and maintain high CD4+ T cell counts despite high levels of virus replication for many years (Viremic Non-Progressors, VNPs) [14]–[17]. Choudhary et al. first reported this rare phenotype in three HIV-infected individuals who maintained high levels of HIV replication (10^4^–10^5^ copies HIV RNA/mL plasma), but had low levels of immune activation and preservation of CD4+ T cells [15]. Rotger et al. subsequently studied a larger cohort of six HIV-infected individuals with persistently high-level viremia who did not show any signs of progressive HIV disease [14]. The VNPs in this study were compared to HIV-infected rapid progressors, and several features of the profile of gene expression (as measured by microarray) were associated with the VNP phenotype, including lower expression of ISGs [14]. In addition, recent studies have demonstrated that VNPs have high levels of mucosal immune activation, corresponding to low levels of mucosal T regulatory cells [18]. Furthermore, VNPs have CD8+ T cell responses similar to those of HIV-infected individuals with chronic disease progression [19]. Interestingly, VNPs share a common gene regulation profile with SIV-infected sooty mangabeys (SMs), an African natural SIV host species that typically experience a non-pathogenic infection despite high virus replication [20] and are thus reminiscent of the rare VNP phenotype. However, it was unclear from these studies whether the differences between VNPs and rapid progressors were simply a consequence of more advanced disease progression in the rapid progressors. For example, HIV-associated immunodeficiency may allow for more microbial translocation and asymptomatic co-infections (i.e. cytomegalovirus, Epstein-Barr virus, etc.), therefore causing greater innate immune activation rather than being a consequence of it [21].

To address these issues, and to identify novel mechanisms that might explain delayed or even lack of disease progression in VNPs, we conducted a detailed immunologic and virologic characterization of a cohort of HIV-infected individuals that were stringently defined as VNPs as previously defined by Rotger et. al [14]. Of note, both the Choudhary and the Rotger studies [14], [15] used as controls HIV-infected individuals with canonical or rapid disease progression. In contrast, in the current study we have used as controls a group of HIV-infected subjects with relatively preserved CD4+ T cell counts. Since VNPs and other LTNPs (including the so-called Elite Controllers, EC) only represent, in aggregate, a very small subset (<1%) of HIV-infected subjects [1], controls with early HIV infection are statistically predicted to progress to AIDS with time if left untreated (hence we termed them “putative progressors”; PP). The rationale for using this type of control group was to avoid the potential confounding effect of immunological impairments that occur in HIV-infected individuals with signs of overt disease progression and complete CD4+ T cell depletion. Here we found that when compared to PP HIV-infected individuals, VNPs showed decreased infection of central memory CD4+ T cells (T_CM_) and stem cell memory T cells (T_SCM_) by HIV, as well as increased proliferation of memory CD4+ T cells, which was associated with increased CD4+ T_CM_ counts. Importantly, T_SCM_ cells have been proposed to represent a preferred viral niche as they characteristically maintain low sensitivity to HIV cytopathic effects and show enhanced long-term survival and proliferation potential as compared to other memory T cell subsets [22], [23], and here we found that the frequency of HIV-infected T_SCM_ cells negatively correlated with the number of T_SCM_ cells. Furthermore, in SIV-infected sooty mangabeys, T_SCM_ are less infected in vivo as compared to pathogenic SIV infection in rhesus macaques [24]. In addition, preservation and decreased infection of T_CM_ in sooty mangabeys has been implicated as a mechanism for protection from disease progression in this model [25]. Thus, the attenuated virus infection in T_CM_ and T_SCM_ cells from VNP individuals may be indicative of at least one mechanism of protection. Taken together, we propose that these novel immunological features of VNPs may underlie maintenance of CD4+ T cells and lack of disease progression in these individuals despite many years of viremic HIV infection.

## Results

### Clinical, virologic, and immunologic characteristics of viremic non-progressors and putative progressors

**Figure 1:**
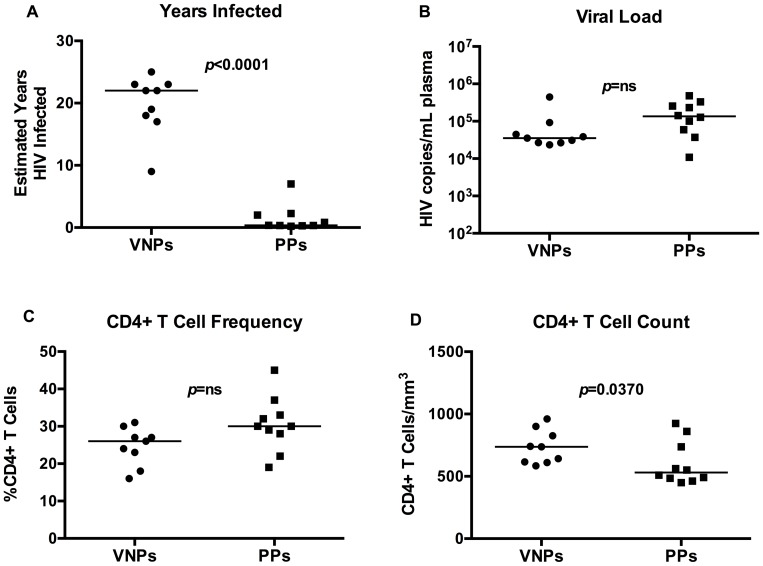
Clinical and immunological characteristics of viremic non-progressors and putative progressors. (**A**) Estimated duration of HIV diagnosis based on first HIV+ test. (**B**) Plasma HIV RNA in VNPs and PPs as measured by RT-PCR. (**C**) The fraction of CD4+ T cells in VNPs and PPs measured by flow cytometry. (**D**) Absolute CD4+ T cell count in blood from VNPs and PPs based on CD4+ T cell fraction and lymphocyte count by CBC. *p* values from Mann Whitney T test. Line reflects median. Circles, VNPs; Squares, PPs.

We identified treatment-naïve HIV-infected VNPs for this study using strict criteria: maintenance of CD4+ T cell counts >500/mm^3^ blood (average 735.0±135.7), sustained plasma HIV RNA levels >10^4^ (average plasma HIV RNA levels 4.68±0.4 log_10_ copies/mL plasma), and >9 years (average 19.8±4.8) since initial HIV diagnosis (Table 1). For comparison, controls were treatment-naïve individuals with confirmed HIV infection (2 months to 7 years since the estimated date of infection [26]), and with comparable levels of viremia (average plasma HIV RNA levels 5.07±0.49 log copies/mL plasma) and relatively comparable CD4+ T cell counts (average 602.9±173.4 CD4+ T cells/mm^3^ blood) (Table 1). These HIV-infected individuals are assumed to progress to AIDS if left untreated at a pace representative of the general HIV-infected population, and were therefore termed “putative progressors” (PP). We chose these controls to minimize confounding by the extent of CD4+ T cell depletion, which could have been a cause rather than a consequence of the immunologic differences observed in prior studies of VNPs. VNPs in our study had been infected for a significantly longer period with a duration of diagnosis of 19.8 years (±4.8) compared to 1.56 years (±02.08) for PPs (*p*<0.0001, Figure 1A). Plasma HIV RNA levels tended to be higher in the PPs, despite the shorter length of infection (*p* = 0.0532, Figure 1B). The percentages of CD4+ T cells among CD3+ T cells were similar between the groups, with a mean of 24.7% CD4+ T cells in VNPs and 30.5% CD4+ T cells in PPs (*p* = 0.0503, Figure 1C), however VNPs maintained a higher absolute CD4+ T cell count than PPs (*p* = 0.0370, Figure 1D). Thus, even though we were successful in sampling HIV-infected controls who had not yet experienced significant peripheral blood CD4+ T cell depletion, the PP controls still had lower CD4+ T cell counts than VNPs.

**Table 1:** Patient characteristics.

VNPs	Sex	Age	Years Infected	CD4 Abs	CD8 Abs	% CD4	% CD8	VL (PCR)
4020	Male	55	23	737	1711	27	62	26,835
1662	Male	58	9	584	1178	23	47	443,248
1208	Male	69	19	960	2054	26	55	30,901
1582	Male	46	18	642	1344	27	57	38,336
1596	Male	46	22	825	2230	24	64	23,364
4015	Male	52	17	741	1367	31	58	44,632
1538	Intersex	54.8	22	611	1989	18	59	26,392
1588	Male	57.3	25	615	2140	16	57	35,200
1319	Male	43	23	900	1737	30	57	92,128

PPs	Sex	Age	Years Infected	CD4 Abs	CD8 Abs	% CD4	% CD8	VL (PCR)
1712	Male	37	2.27	509	807	30	47	59,488
1836	Male	22	0.36	491	1374	22	61	478,764
1847	Male	31	0.20	737	857	33	38	128,031
1845	Female	42	Unknown	551	803	29	43	37,025
1831	Male	48	0.28	462	1535	19	65	142,366
1793	Male	42	0.84	484	1021	28	59	232,177
1374	Male	34	0.34	860	630	45	33	253910
1469	Male	31	0.34	924	1509	30	49	100932
1723	Male	29	2	562	761	32	43	10886
4010	Male	45	7	449	559	37	46	331000

### VNPs have increased levels of T stem cell memory cells compared to PPs

**Figure 2:**
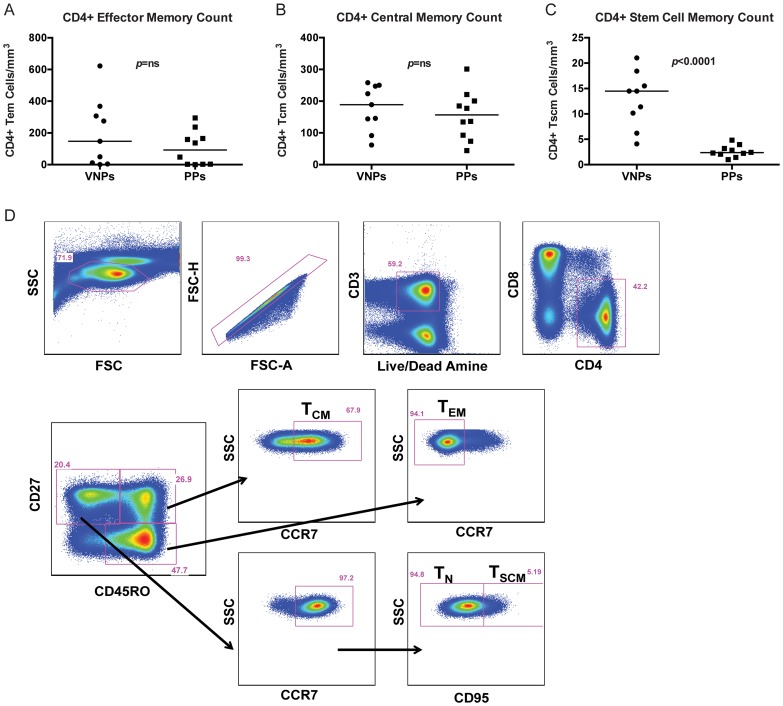
CD4+ T cell memory counts and representative flow cytometry staining. (**A–C**) The absolute number of CD4+ T cell subsets in VNPs and PPs was measured by flow cytometry and lymphocyte count by complete blood cell (CBC) counts. (**A**) The absolute number of CD4+ T_EM_ cells; (**B**) The absolute number of CD4+ T_CM_ cells; (**C**) The absolute number of CD4+ T_SCM_ cells in VNPs and PPs. (**D**) Representative flow cytometry staining for each CD4+ T cell memory subset. *p* values from Mann Whitney T test, line reflects median. Circles, VNPs; Squares, PPs.

We next sought to determine whether CD4+ memory T cell populations were preserved in VNPs. We measured central memory CD4+ T cells (T_CM_; live, singlet, CD3^+^CD4^+^CD27^+^CD45RO^+^CCR7^+^ cells), stem cell memory CD4+ T cells (T_SCM_; live, singlet, CD3+CD4+CD27+CD45O-CCR7+CD95+ cells), naïve CD4+ T cells (T_N_; live, singlet, CD3+CD4+CD27+CD45O-CCR7+CD95− cells) and effector memory CD4+ T cells (T_EM_; live, singlet, live, singlet, CD3^+^CD4^+^CD27^−^CD45RO^+/dim^CCR7^−^ cells) based on previous studies [23] (representative flow cytometry staining Figure 2D). We found that there was no significant difference in the number of CD4+ T_EM_ cells (Figure 2A) or CD4+ T_CM_ (Figure 2B) in VNPs compared to PPs. However, the number of CD4+ T_SCM_ cells were significantly depleted in PPs compared to VNPs (*p*<0.0001, Figure 2C), demonstrating that these important regenerative memory cells are preserved in VNPs.

### VNPs have similar levels of immune activation compared to PPs but increased proliferation of CD4+ memory T cells

**Figure 3:**
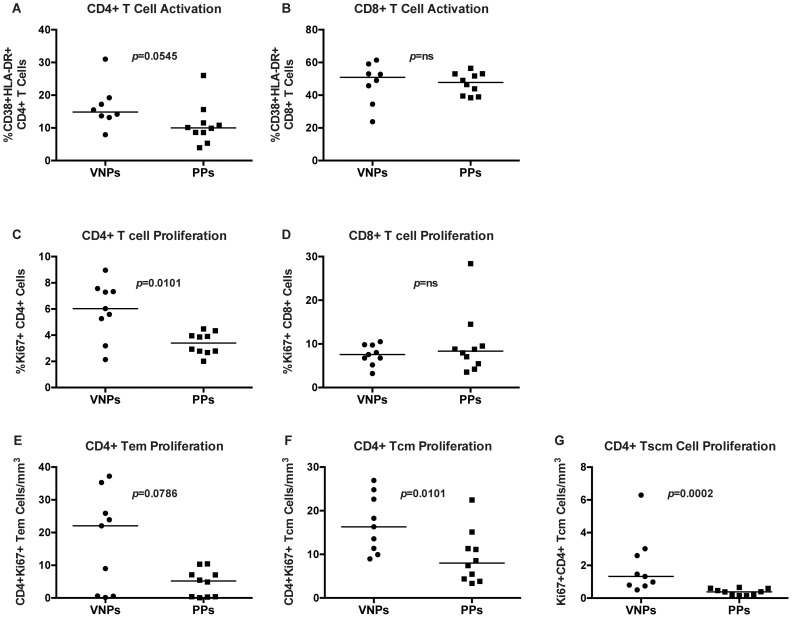
T cell proliferation and activation in VNPs and PPs. (**A–B**) The frequency of activated CD38+HLA-DR+ T cells in VNPs and PPs by flow cytometry: (**A**) total CD4+ CD38+HLA-DR+ T cells and (**B**) CD8+ CD38+HLA-DR+ T cells. (**C–D**) The frequency of proliferating (Ki67+) VNPs and PPs by flow cytometry: (**C**) total CD4+Ki67+ T cells and (**D**) total CD8+Ki67+ T cells. (**E–G**) The absolute count of Ki67+CD4+ T cell memory subsets measured by flow cytometry and lymphocyte count by CBC for: (**E**) CD4+Ki67+ T_EM_ cells; (**F**) CD4+Ki67+ T_CM_ cells; (**G**) and CD4+Ki67+ T_SCM_ cells. *p* values from Mann Whitney T test. Line reflects median. Circles, VNPs; Squares, PPs.

To assess whether lack of disease progression in VNPs compared to PPs was associated with decreased immune activation, we measured the frequencies of CD38+HLA-DR+ T cells in peripheral blood. Indeed, expression of the markers CD38 and HLA-DR is increased during chronic HIV infection and correlates with disease progression [27]. Unexpectedly, we found similar levels of T cell activation in VNPs compared to our PP cohort in both CD4+ and CD8+ T cells (Figure 3A–B). To investigate further how VNP maintain normal CD4+ T cell counts despite active virus replication, we next evaluated Ki67 expression, an index of cellular cycling/proliferation. We found that the frequency of total CD4+ T cells that expressed Ki67 was significantly higher in VNPs than PPs in bulk CD4+ T cells (*p* = 0.0101, Figure 3C), but there was no difference in bulk CD8+ T cell proliferation (Figure 3D).

We further assessed proliferation in CD4+ T cell memory subsets by measuring expression of Ki-67 in memory CD4+ T cell subsets (T_EM_, T_CM_, and T_SCM_). We observed a non-significant trend (*p* = 0.0786, Figure 3E) towards higher frequencies of Ki-67+ CD4+ T_EM_ from VNPs compared to levels in PPs. However, we found significantly higher frequencies of Ki-67+ CD4+ T_CM_ and T_SCM_ cells in VNPs compared to PPs (*p* = 0.0101, Figure 3F and *p* = 0.0002 Figure 3G respectively). Thus, overall, CD4+ T cells in VNPs had increased proliferation compared to PPs.

### VNP show decreased levels of cell associated HIV-DNA in CD4+ T_SCM_ and T_CM_

**Figure 4:**
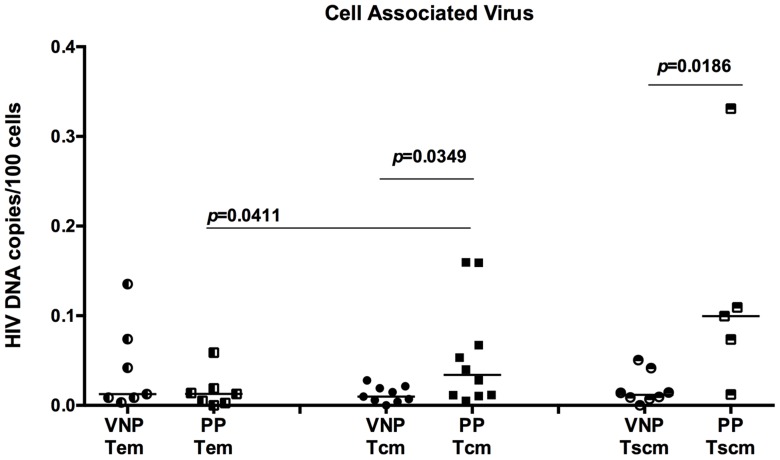
VNPs have decreased HIV infection of central memory and stem cell memory T cells. CD4+ T_EM_ cells (left), T_CM_ (center), and T_SCM_ (right) from VNPs and PPs were sorted by flow cytometry and quantitaive real-time PCR was used to determine the HIV infection frequency in each subset. Infection frequency was determined by copies of *gag* DNA/100 infected cells. *p* values from Mann Whitney T test (VNPs vs PPs) or paired T test (T_CM_ vs T_EM_ within PP cohort). Line reflects median. Circles, VNPs; Squares, PPs.

The observation of increased frequencies of Ki-67+ CD4+ T_CM_ and T_SCM_ in VNP raises the possibility that these cells may be potential targets for virus infection as a result of their proliferation state. To directly test this possibility, we next determined the number of HIV DNA copies in flow cytometrically sorted CD4+ T cell subsets, including CD4+ T_SCM_, T_CM_, and T_EM_. We assessed HIV DNA copies by quantitative real time PCR as described in [28]. The levels of viral DNA in CD4+ T_EM_ were similar in both groups (*p* = 0.4458, Figure 4 left). However, despite over a decade longer duration of untreated HIV infection and higher frequencies of cycling CD4+ T cells, VNPs had significantly lower levels of cell associated HIV DNA in their CD4+ T_CM_ than in CD4+ T_CM_ from PPs (*p* = 0.0349 Figure 4 middle), as well as decreased levels of HIV DNA in T_SCM_ in VNPs compared to PPs (*p* = 0.0186, Figure 4 right). In addition, while PP have significantly increased infection of T_CM_ compared to infection in T_EM_ (*p* = 0.0411, Figure 4), there was not a significant difference between the HIV DNA in T_CM_ and T_EM_ of VNPs. These data are strikingly consistent with the observed low levels of SIVsmm DNA in CD4+ T_CM_ during non-progressive infection of sooty mangabeys and in long-term non progressors [9], [25], and suggest that a possible common mechanism underlying the lack of disease progression in HIV-infected VNP, LTNPs, and SIVsmm-infected SMs is the relative resistance of CD4+ T_CM_ from direct virus infection. In addition, VNPs also had decreased infection of T_SCM_ cells, which have recently been described to be essential for long-lived memory reservoirs, and are preferentially infected in progressive HIV infection [22], [23]. To our knowledge, T_SCM_ infection has not been assessed in other non-progressive cohorts, however, preservation of long-lived memory CD4+ T cells is likely essential for lack of disease progression in VNPs.

### Differential mechanisms may underlie survival of CD4 memory T cell populations in VNPs

**Figure 5:**
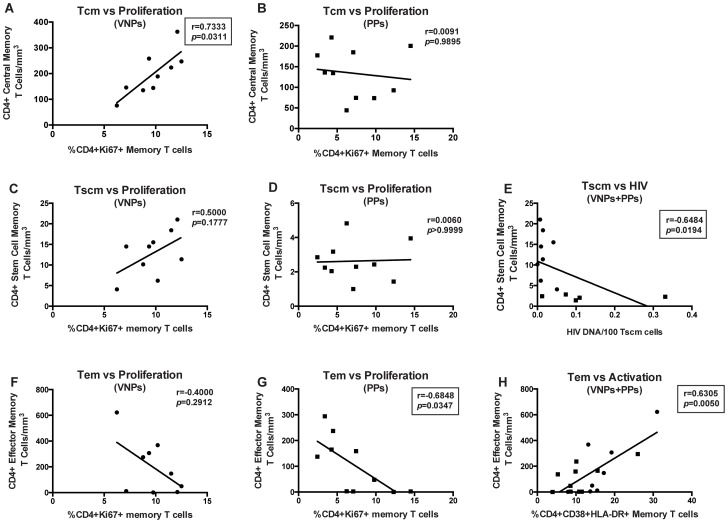
CD4+ T cell memory subset numbers are associated with differential preservation mechanisms. CD4+ T_CM_ cells (**A–B**): Correlation between frequency of CD4+Ki67+ memory cells and absolute number of CD4+ T_CM_ cells in VNPs (**A**) and PPs (**B**). CD4+ T_SCM_ cells (**C–D**) Correlation between frequency of CD4+Ki67+ memory cells and absolute number of CD4+ T_SCM_ cells in VNPs (**C**) and PPs (**D**). (**E**) Correlation between HIV DNA infection per 100 CD4+ T_SCM_ cells and absolute number of CD4+ T_SCM_ cells. CD4+ T_EM_ cells (**F–H**): Correlation between frequency of CD4+Ki67+ memory cells and absolute number of CD4+ T_EM_ cells in VNPs (**F**) and PPs (**G**). (**H**) Correlation between frequency of CD4+CD38+HLA-DR+ memory T cells and absolute number of CD4+ T_EM_ cells. *p* and r values from spearman correlations, with linear regression shown as line. Box around significant (*p*<0.05) values. Circles, VNPs; Squares, PPs.

We next sought to determine what mechanisms may underlie protection of CD4+ memory T cell populations. However, differences were apparent when comparing the number of T_CM_ to the frequency of proliferating memory CD4+ T cells. Indeed, in VNPs, we found a significant positive correlation between the frequencies of CD4+Ki67+ memory cells compared to the number of T_CM_ cells (r = 0.7333, *p* = 0.0311, Figure 5A). However, in PPs, this correlation did not exist (r = 0.0091, *p* = 0.9895, Figure 5B), suggesting that proliferation of T_CM_ cells may underlie disease protection after several years of HIV infection in VNPs, but not in PPs, who will ultimately have depletion of these cells if left untreated. This suggests that an increased and/or more efficient proliferation of CD4+ T_CM_ may be a mechanism underlying preserved CD4+ T cells after several years of infection, despite active virus replication. While a similar trend as T_CM_ existed between T_SCM_ cells and frequency of proliferating CD4+ memory cells in VNPs, it did not reach significance (r = 0.5000, *p* = 0.1777, Figure 5C), while the lack of association was consistent in PPs (*p*>0.9999, Figure 5D). Given the lack of a correlation between proliferation and the number of T_SCM_ cells, despite the preserved nature of these cells in VNPs, we investigated whether the decreased HIV infection in T_SCM_ cells was associated with the preservation of these cells. Indeed, we found a significant, negative correlation between the frequency of HIV-infected T_SCM_ cells and the overall number of T_SCM_ cells (r = −0.6484, *p* = 0.0194, Figure 5E). However, of note, this relationship between HIV infection and cell count did not exist in the T_CM_ cell subset (r = −0.2246, data not shown). Thus, these data indicate that a potential mechanism underlying preservation of T_SCM_ cells in VNPs compared to PPs is decreased HIV infection of these essential long-lived cells.

Finally, we assessed the role of CD4+ effector memory T cells (T_EM_), and found that T_EM_ cells had the opposite relationship with proliferation, with a trend towards a negative correlation between T_EM_ cells and proliferating CD4+ memory cells in VNPs (r = −0.4000, *p* = 0.2912 Figure 5F), and a significant negative correlation between T_EM_ cells and proliferating CD4+ memory T cells in PPs (r = −0.6848, *p* = 0.0347, Figure 5G). Furthermore, we observed a significant correlation between the frequency of activated (CD38+HLA-DR+) CD4+ T memory cells and T_EM_ cells in both cohorts (r = 0.6305, *p* = 0.0050, Figure 5H). However, no relationship was observed between T_EM_ cells and HIV infection of T_EM_ cells (r = −0.0506, data not shown), nor did we observe any relationship between CD4+ T cell memory activation and the frequency of T_CM_ or T_SCM_ cells (*p* = 0.4365 and *p* = 0.2611, respectively, data not shown). Taken together, these data indicate that different mechanisms may underlie preservation of CD4+ T cells in VNPs despite several years of infection and high virus replication, with proliferation mainly associated with T_CM_ cells, lack of HIV infection associated with T_SCM_ cells, and activation driving T_EM_ cells.

### Transcriptome analysis of viremic non-progressors

**Figure 6:**
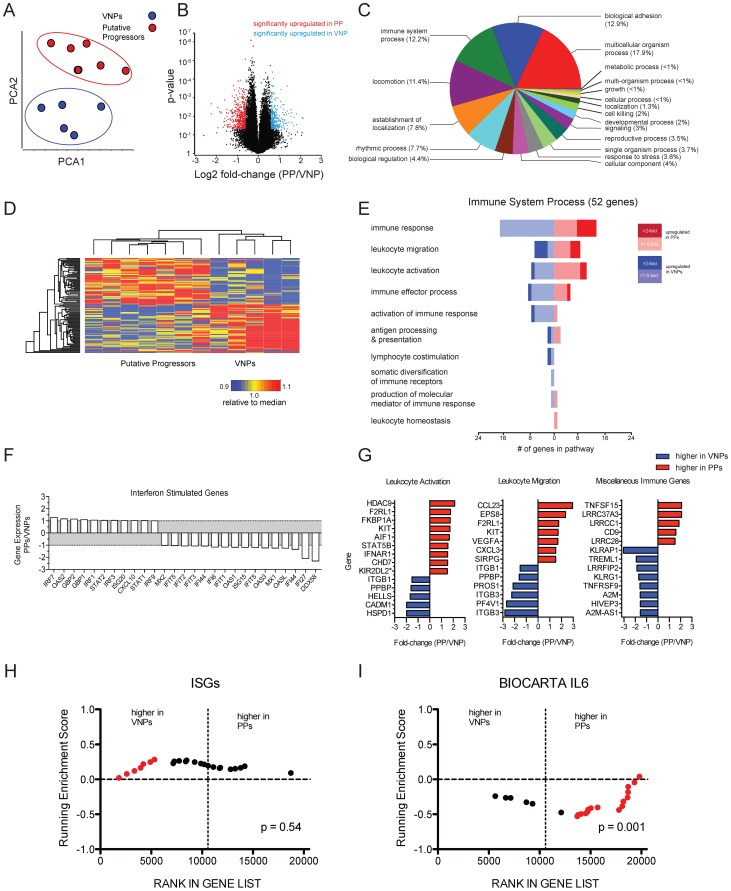
Transcriptomic signatures between HIV-infected putative progressors and viremic non-progressors. Gene expression profiles from whole blood were assayed by microarray analysis. (**A**) Prinicipal components analysis was used to assess the divergence of the entire transcriptomic profile of the patient classes. (**B**) Volcano plot showing the distribution of fold-changes and significance level between the PP and VNP phenotypes and the *p*-value assessed by two-sample t-test. Red data points indicate genes upregulated in PP patient samples, defined as >1.5-fold greater than the VNP samples, and a *p*-value of <0.05. Blue data points indicate genes >1.5-fold in VNPs with 0.05 p-value. The y-axis is plotted as the log of the inverse of the p-value. (**C**) Pathway analysis of genes differentially expressed between VNPs and PPs was performed using the Gene Ontology database. Fisher's exact test was used to estimate the significance of enrichment an annotations of probesets on the arrays were used as a background. The forest plot shows genes identified by GO as have immune function and divides them into GO subcategories. (**D**) Heat map showing the overall concordance amongst patients in each class. The top 50 most highly expressed genes were median-normalized and organized by hierarchical clustering using Pearson's dissimilarity metric and average linkage. (**E**) Forest plot of 52 genes defined in the GO category “Immune Process” in panel C. Branches indicate subcategories, the number of genes with higher expression in each phenotype are indicated by the scale on the x-axis, and the magnitude of the fold-change is indicated by the color scale. (**F**) Relative expression of ISGs between phenotypes. (**G**) Forest plots of individual genes contained with GO categories and unincorporated immune genes. *probeset is predicted to hybridize to multiple genes. (**H & I**) Gene Set Enrichment plots of ISGs and IL6 signaling genes. GSEA performed using geneset permutation, and array data ranked by signal-to-noise ratio. Vertical dotted lines indicate the margin between gene upregulated in VNPs (left) and PPs (right) of all non-redundant, annotated genes on the array. Data points in red indicate leading edge genes contributing to the majority of the enrichment score.

To further identify potential mechanisms underlying the VNP phenotype, we performed microarray analysis on RNA derived from whole blood from five VNP and seven PP HIV-infected individuals. Similarity of the transcriptomic profiles of individual patients was visualized using Principal Components Analysis (PCA). PCA indicated that the total transcriptomic profiles clustered according to their status as either a VNP or PP (Figure 6A) indicating that there was a conserved transcriptomic signature specific to each patient cohort. Genes differentially regulated within each patient class were defined as those displaying a fold-change of greater than +/−1.5 relative to the reciprocal class, and with an unadjusted *p*-value of *p* = 0.05 defined by two-sample T-test. Using these criteria, we defined 769 probesets (476 higher in VNPs compared to PPs, 293 higher in PPs reciprocally) representing 499 annotated transcripts that were differentially expressed between subsets (listed in Tables S1 and S2, respectively). The distribution of genes upregulated in each class using these criteria are depicted in Figure 6B. Hierarchical clustering demonstrated that the genes were consistently expressed across individuals within each patient class (Figure 6D). To summarize biological functions represented within the differentially expressed genes, we tested for enrichment of pathways annotated within the Gene Ontology (GO) database (Figure 6C). The pathways demonstrating the highest level of enrichment were those regulating immune processes and cellular motility pathways (biological adhesion, locomotion, establishment of localization). 52 genes within these pathways were annotated as having immune function, and we further sub-classified these genes according to their expression levels in PPs and VNP (represented in the forest plot in Figure 6E). The largest number of genes were in the category ‘immune response”, “leukocyte migration” and “leukocyte activation” –Figure 6G demonstrates individual activation-related genes higher in either phenotype: PPs had elevated expression of IFNAR1, which encodes for one chain of the Type I IFN receptor, a probeset specific for KIR2-transcripts. We also noted elevated expression of STAT5B in PPs, which signals downstream of IL7, a cytokine important for maintaining T cell homeostasis (STAT5B was also the lone gene comprising the “leukocyte homeostasis” category); however we were not able to detect significant enrichment genes downstream of IL7. In addition, cytokine and chemokine analysis in plasma by luminex demonstrated that neither IL-7, nor any other cytokines and chemokines measured, were significantly different in VNPs compared to PPs (data not shown).

In the “leukocyte migration” category, we noted expression of two chemokines: two independent probesets for CCL23 were upregulated in PPs (data from one probeset shown in Figure 6G). CCL23 is highly homologous to CCL3/MIP1A, however acts as an agonist for CCR1 and has been shown to induce signaling in monocytes and resting T cells, but has not been studied in the context of HIV infection [29]. CXCL3, which has been demonstrated to inhibit proliferation and induce apoptosis in T cells, was also upregulated in PPs [30]. There were a number of genes that were not incorporated into the GO annotation that have putative immune function (depicted in “miscellaneous” in Figure 6G). Upregulated in PPs was TNFSF15/TL1A, a broadly activating co-stimulatory molecule from the TNF-superfamily associated with several inflammatory disorders (Figure 6G). TNFSF15/TL1A ligation of its cognate receptor, DR3, has been demonstrated to induced apoptosis in T cell lines in vitro and primary endothelial cells [31], and recently was implicated in inducing inflammatory expression in CD4+CD161+ T cells in gut inflammation [32]. Also interesting were several leucine-rich repeats which had higher expression in VNPs, and which were recently demonstrated to inhibit activation of the NLRP3 inflammasome [32].

In previous work, we performed a microarray analysis of CD4+ and CD8+ T cells isolated from VNP and found that interferon stimulated genes (ISGs) were expressed at higher levels in chronically infected rapid progressor patients compared to VNPs, despite higher viral loads in the latter patient [14]. While a handful of ISGs had increased expression in VNP or PP (Figure 6F), the majority of probes representing ISGs did not display any significant variation, nor did they exhibit enriched expression in either phenotype (Figure 6H), despite the elevated expression of IFNAR1 in the PPs (Figure 6G). To increase the sensitivity of detecting molecular pathways differentially regulated between patient groups, we used gene set enrichment analysis (GSEA) [33], and confirmed that there was not a consistent upregulation of Type I interferon between PPs and VNPs (Figure 6H).

We next used GSEA to test for the enrichment of transcripts involved in other immune activation pathways. To identify other potential pathways of immune activation, we selected six genesets from MSIGDB that are related to immune activation and inflammation. Of note, we noted that the pathway representing canonical IL6 signaling (BIOCARTA ID:M5489) was significantly enriched in the PP phenotype (Figure 6I). Further analysis demonstrated that multiple genesets representing overlapping but distinct genes implicated in IL6 signaling were enriched in the PP phenotype relative to VNPs. Within the microarrary data, IL6 had very low but consistent upregulation in PPs (fold-change = 1.18, *p* = 0.006, data not shown). The observation of multiple genesets demonstrating consistent enrichment in IL6 signaling indicates that PP patients have overall elevated expression of genes in this pathway. However, no differences in IL-6, or any other cytokines or chemokines measured, were observed in the plasma between PP and VNP patients (data not shown). Thus, the differences in cytokine signaling and/or leukocyte activation observed by microarray may reflect overt immunological dysfunction in the PPs compared to the VNPs, consistent with progressive HIV phenotype [34], [35].

## Discussion

Viremic Non-Progressors (VNPs) are infrequent among HIV-infected individuals and remain clinically asymptomatic and maintain high CD4+ T cell counts despite many years of infection with robust virus replication. Interestingly, these VNPs show striking similarities with SIV-infected sooty mangabeys (SMs), an African “natural” host non-human primate species whose infection is typically non-pathogenic and characterized by healthy CD4+ T cell counts and low immune activation despite high levels of virus replication (Reviewed in [36]). Earlier reports [14], [15], [18], [19] compared VNPs to HIV-infected individuals who had been infected longer, with much greater degrees of CD4+ T cell depletion. To address this issue, we studied a group of stringently defined VNPs and, in contrast to previous studies, we compared them to HIV-infected individuals with similar CD4+ T-cell counts and viral load, mainly in early infection (i.e. “putative progressors”; PP). Of note, comparing subjects mainly in early infection has the potential caveat that the results are influenced by length of infection. However, there is no correlation between years infected and infection frequency in any of the CD4+ T cells subsets measured (data not shown). In addition, previous studies have demonstrated that chronically infected individuals have high levels of HIV DNA and/or reservoir in T_CM_ and T_SCM_ cells, and increased HIV DNA and RNA compared to recently infected individuals, supporting the concept that this comparative cohort of PPs is appropriate [22], [37]–[39].

Here we demonstrated that VNPs have decreased infection of both stem cell memory and central memory CD4+ T cells by HIV, and also had increased frequencies of Ki-67+ CD4+ T cells. Because the expression of Ki-67 was higher in VNPs than in PPs, while immune activation markers such as HLA-DR and CD38 were expressed at similar levels, we propose that, in VNPs, the combination of increased CD4+ T memory cell proliferation and low levels of direct virus infection of CD4+ T_SCM_ and T_CM_ cells may be reflective of more efficient homeostatic proliferation, a physiologic process induced by loss of lymphocytes, rather than overt activation. Indeed, in non-pathogenic infection of SMs, there is a similar association between T_CM_ count and proliferation [40]. However, whether these factors represent a cause or consequence of lack of progression in VNPs and altered T_CM_ and T_SCM_ infection is unclear. Indeed, in VNPs, proliferation of CD4+ T_CM_ was associated with increased numbers of CD4+ T_CM_, while the opposite was true in PPs. And while a similar trend existed in T_SCM_ cells, the strongest association with preservation of T_SCM_ cells in VNPs is their lower frequency of HIV infection. Of note, the absolute number of CD4+ T cells that are infected T_CM_ in VNPs compared to PPs loses significance, but maintains a trends towards decreased infection (*p* = 0.0999, Figure S2). However, a significant difference between VNPs and PPs is maintained when the absolute number of T_SCM_ cells that are infected is calculated (*p* = 0.0295, Figure S2).

In this study, we found that VNP HIV-infected individuals were remarkably similar to PPs in terms of markers of systemic immune activation as well as overall profile of gene expression. This finding seemingly is in contrast to previous studies suggesting that VNPs had low immune activation and a profile of gene expression characterized by low ISG expression [14], [15]. However, we believe that the choice of PP HIV-infected individuals as controls allowed us to identify the immunological features of VNPs that distinguish this rare patient population, avoiding confounding by progressive infection. Collectively, the transcriptomic data have identified that VNPs have elevated expression of several genes associated with immune homeostasis, and conversely, lower expression of inflammatory genes compared to non-VNPs. However, given the disparate associations we observed between CD4+ memory cell subsets and activation (T_EM_ cells), proliferation (T_CM_ cells) and HIV infection (T_CM_ cells), RNA studies in whole blood as was performed here may not be as informative, and future studies that sort memory subsets would be of great interest. While these data provide only an associative link between the identified candidates and the VNP phenotype, in combination with the immunophenotyping data, the gene expression adds further support to a model in which HIV infection in VNPs is associated with lower inflammation. In this regard, the current study suggests that the low immune activation and absence of significant ISG up-regulation in VNPs could potentially be a consequence rather than a cause of their preserved immune status.

One potential explanation of the VNP phenotype is that these individuals are infected with HIV-1 strains that show intrinsically lower infectivity and/or cytopathicity compared to strains isolated from normal progressors. However, the study by Choudhary et al. indicated that, in organ culture, HIV isolates derived from VNP were as cytopathic as viruses isolated from normal HIV progressors [15]. However, we performed a supplemental study in an additional group of HIV-infected individuals, including several individuals with a phenotype similar to viremic non-progressors (Table S3) to investigate the accessory Nef protein. It has been previously demonstrated that the efficient suppression of T cell activation and apoptosis by Nef-mediated down-modulation of TCR-CD3 may help the infected host to prevent chronic immune activation and CD4+ T cell depletion [41]. However, just like *nef* alleles from HIV-1-infected individuals with progressive infection, those derived from VNPs were generally unable to remove CD3 from the cell surface (Figure S1). Overall, the differences in Nef function between VNP and chronic progressor HIV-infected individuals were much more subtle than those established for HIV-1 and SIVsmm Nefs [41], and it is unclear whether differences in Nef function are a cause or consequence of differences in disease progression.

An additional potential mechanism of protection from disease progression in VNPs is CD8+ T cell mediated immunity. In our analysis, while we observed an increase in CD8+ T cell count in VNPs, we did not find an increase in proliferation or associations between CD8+ T cell subsets and proliferation, or HIV levels in CD4+ T cells as we observed for CD4+ T cells (data not shown). In addition, given that virus load is not controlled in plasma, overall CD8+ T cell control is unlikely, and previous studies of viremic controllers demonstrated that CD8+ T cell immunity was not increased [19]. However, in long-term non-progressors with low viremia, HIV-specific CD8+ T cell responses are associated with limited T_CM_ infection, particularly in HLA-B27 and HLA-B57 patients [9]. Indeed, a potential mechanism may exist whereby CD8+ T cells can mount preferential protection against T_CM_ and T_SCM_ infection, and this possibility should be investigated in future work. In addition, while we saw no significant difference in the expression of CCR5 on CD4+ T cells subsets between VNPs and PPs in this study, the role of HIV co-receptors in protection from infection in VNPs should be further investigated. Lastly, another possible mechanism for protection is differential expression of restriction factors in CD4+ T cells subsets of VNPs. Indeed, understanding the mechanisms by which these cells are protected will be crucial in understanding the lack of progression and potential intervention strategies.

The observation that VNPs have significantly lower infection of both CD4+ T_CM_ and T_SCM_ than do the same subsets in PPs identifies a novel, potentially crucial mechanism of protection of CD4+ T cell homeostasis in this rare subset of HIV-infected individuals. In addition, it identifies another striking similarity between VNPs and naturally SIVsmm-infected SMs, who also experience a non-pathogenic, immunologically benign infection despite chronic virus replication [20]. Our observation that T_CM_ and T_SCM_ in VNPs harbor less HIV DNA as opposed to PPs is also consistent with another recent report suggesting that VNPs tend to have lower T cell activation than progressors in peripheral blood, yet higher T cell activation in the rectal mucosa, where a much higher proportion of CD4+ T cell have an effector phenotype [18]. Preservation of CD4+ T_CM_ and T_SCM_ from direct virus infection may be of particular importance during HIV and SIV infections, as these cells are longer lived than CD4+ T_EM_, and proliferation of T_SCM_ feeds the CD4+ T_CM_ cell pool, which in turn is essential to maintain a sufficient number of CD4+ T_EM_ in mucosal tissues [42]. Indeed, previous studies by Okoye et al. have elegantly shown that while CD4+ T_EM_ depletion is the proximate mechanism of immunodeficiency, the tempo of SIV disease progression is largely determined by destruction, failing production, and gradual decline of CD4+ T_CM_ cells [42]. Thus, a shared mechanism based primarily on preserving CD4+ T_SCM_ and T_CM_ cells from virus infection may underlie the lack of disease progression in both VNPs and SIVsmm-infected SMs.

Finally, emerging data suggest T_SCM_ cells represent an important niche for replication-competent viral reservoir, especially given their ability to harbor immense amounts of virus when measured on a per cell basis [22]. T_SCM_ cells stably persist in secondary lymphoid organs and provide multipotent and self-renewing potential which allows for the incorporation of abundant virus into other T cell memory phenotypes downstream of proliferating T_SCM_ cells [22], [23]. Thus, future studies to determine possible mechanisms underlying T_CM_ and T_SCM_ cell resistance to direct virus infection, such as genetic factors, co-receptor regulation, restriction factor expression and viral determinants may provide critical information to better understand how VNPs avoid CD4+ T cell loss and maintain attenuated disease progression.

## Methods

### Patient samples

HIV-infected viremic non-progressor (VNP) and putative progressor (PP) samples were sampled from the UCSF SCOPE and OPTIONS cohorts, respectively. VNPs were defined as having confirmed HIV-1 infection for more than 9 years with sustained plasma HIV RNA levels >10,000 copies/ml and maintenance of peripheral blood CD4+ T cell counts >500 cells/mm^3^ and a CD4% (of all lymphocytes) >15% (Table 1). Recently HIV-infected PPs were defined as having plasma HIV RNA levels >10,000 copies/mL, CD4+ T cell counts >400 cells/mm^3^ and having been initially infected with HIV 2 months to 7 years prior to the index visit (Table 1). The estimated date of initial HIV infection was calculated according to published algorithms that incorporate “de-tuned” anti-HIV-1 antibody ELISA results [43], [44] or by a documented sero-conversion window of <6 months. All participants were required to be antiretroviral therapy (ART)-naïve.

### Immunophenotyping

Cryopreserved PBMCs were isolated from whole blood, and stored at the UCSF AIDS Specimen Bank. T cell activation was measured by the UCSF Core Immunology Laboratory, as previously described and optimized [45]. Cryopreserved PBMCs were thawed and stained with the following markers: Aqua Amine Reactive Dye (Invitrogen, Carlsbad, CA), CD3 Pacific Blue, CCR5 PE-CY5 (BD Pharmingen, San Jose, CA), CD38 PE, HLA-DR FITC, (BD Biosciences), CD4 PE Texas Red, and CD8 QDot 605 (Invitrogen).

### Cell sorting

CD4^+^ T cells were sorted into T_CM_ and T_EM_ subsets using a BD FACS Aria (BD Biosciences, San Jose, CA) run by BD FACS DIVA software. T_CM_ were defined as live, singlet, CD3^+^CD4^+^CD27^+^CD45RO^+^CCR7^+^ cells. T_EM_ were defined as live, singlet, CD3^+^CD4^+^CD27^−^CD45RO^+/dim^CCR7^−^ cells, T_SCM_ were defined as live, singlet, CD3+CD4+CD27+CD45O-CCR7+CD95+ cells as previously described and as demonstrated in Figure 2D [23]. Cryopreserved PBMCs were thawed and stained with predetermined optimal concentrations of the following markers: Aqua Amine Reactive Dye (Invitrogen), CD3 AL700, CD8 PERCPCY5.5, CCR5 PE, CCR7 PE-CY7, CD95 PE-CY5 and Ki67 FITC (BD Pharmingen), CD4 eFluor450, CD27 APC-eFluor780 (eBiosciences, San Diego, CA), CD45RO ECD (Beckman Coulter, Chaska, MN).

### Cell associated virus

HIV DNA was quantified in CD4+ T_CM_ and T_EM_ subsets using an ABI StepOnePlus real time PCR system (Life Technologies, Grand Island, NY) as previously described [28], [46]. Albumin was used to determine cell number in each reaction, and *gag* DNA was used simultaneously quantified to determine HIV levels with HIV *gag* forward primer: GGTGCGAGAGCGTCAGTATTAAG; HIV *gag* reverse primer: AGCTCCCTGCTTGCCCATA; and HIV *gag* probe: AAAATTCGGTTAAGGCCAGGGGGAAAGAA. Duplicate reactions were run and template copies were calculated with ABI software. If no viral DNA was amplified from a given cell population, we report half the lower limit of detection, based on twice the number of cells put into each PCR as previously described [28].

### RNA preparation and microarray hybridization

RNA extraction and microarray analysis were conducted at the Yerkes NHP Genomics Core Laboratory (http://www.yerkes.emory.edu/nhp_genomics_core/). Whole blood was from HIV-infected donors was collected into RNA PAXgene tubes (QIAGEN, Valencia, CA) and purified as previously described [47]. Purified RNA was assessed by Nanodrop and Agilent Bioanalyzer analysis; all samples had RIN scores>8.0. 100 ng of total RNA was amplified, labeled and hybridized to Affymetrix Human U133 Plus 2.0 arrays (Affymetrix, Santa Clara, CA) using the NuGEN Ovation RNA Amplification System V2, Ovation WB Reagent and Encore Biotin Module according to manufacturer's specifications (NuGEN Inc, San Carlos, CA). After hybridization, arrays were washed on Affymetrix FS450 fluidics stations using the NIRAV-WASH protocol and scanned on an Affymetrix 3000 7G GeneChip Scanner.

### Microarray data analysis

CEL files from individual arrays were preprocessed and normalized by RMA within PARTEK Genomics Suite Software. NUSE and RLE plots were inspected to ensure there were no outlier arrays at the hybridization level. PCA was on the RMA normalized data and determined one VNP patient to be an outlier from both VNPs and PPs, and was removed from downstream statistical analysis. To determine differentially expressed genes, a two-tailed T-test was run using Partek Genomics Suite software (v.6.13, Partek Inc, St. Lousi MO). Using Benjamini-Hochberg correction for multiple hypothesis testing yielded only two unannotated transcripts detected as differentially expressed between patients. Several genes within the dataset were upregulated or downregulated several-fold, with consistent expression values across multiple redundant probesets, suggesting the BH corrected *p*-value (1.8e-06) was overly stringent. To prioritize genes with differential expression, we relaxed our gene-filtering criteria to an unadjusted *P* -value of *P* = 0.05 and a fold-change of +/−1.5 between classes. Lists of genes with differential expression between classes using this criteria are contained in Table S1 (elevated in VNPs) and Table S2 (elevated in PPs). Gene Set Enrichment Analysis (GSEA) was used as a more sensitive method to detect significantly enriched pathways in either the VNP or PP transcriptomes. GSEA software was downloaded (http://www.broadinstitute.org/gsea/index.jsp) and run locally using the following parameters: Signal2Noise metric for ranking genes; the dataset and genesets were converted into Gene Symbols; 1000 geneset permutations; redundant probesets were collapsed using the max probeset and the ‘weighted’ enrichment statistic was employed. Microarray data was submitted to the GEO database according to MIAME standards.

### Statistical analysis

Statistical calculations were performed as follows: (i.) Mann-Whitney non-parametric *t* test for comparison of VNPs to PPs; (ii.) Paired *t* test for comparison of VNPs to VNPs and PPs to PPs; (iii.) Spearman correlation with linear regression was used for all correlative analysis. All statistical analysis was performed using Graph Pad Prism, version 5.0. *P* values of <0.05 were considered significant for *nef* analysis.

### Accession numbers

The raw data for the microarray analysis has been deposited at the NCBI GEO database (http://www.ncbi.nlm.nih.gov/geo/) under accession #GSE57730.

### Ethics statement

All SCOPE and OPTIONS cohort samples (Table 1) were obtained after written informed consent and approval by the University of California San Francisco Institutional Review Board. All BRESCIA cohort patient samples (Supporting data) were obtained after written informed consent and approval by the University of Brescia Institutional Review Board.

## Supporting Information

Figure S1 **Functional characterization of** ***nef*** **alleles from VNPs and VPs.** *Nef* functions were characterized in a separate cohort of VNPs and chronically HIV-infected viremic progressors (VPs). (**A**) Quantitation of Nef-mediated down-modulation of CD4, MHC-I, CD28 and CD3 on PBMCs infected with HIV-1 Nef/eGFP constructs. HIV-1 *nef* genes were grouped based on the patient characteristics and those from VNPs are color coded green. Each symbol indicates the *n*-fold down-modulation of the indicated receptor molecule by one of the 26 different NL4-3 proviral constructs. (**B**) Nef-mediated enhancement of virion infectivity. P4-CCR5 indicator cells were infected with proviral constructs expressing patient-derived *nef* alleles. Infections were performed with virus stocks containing 1 ng p24 antigen. Values represent the averages of two experiments compared to the infectivity of the virus expressing the NL4-3 Nef (100%). Values in panels A and B represent averages from two or three experiments, and the horizontal bars indicate average activities per group. The results obtained for the HIV-1 Nef/eGFP control constructs are indicated by lines: red, NL4-3 *nef*; broken blue, SIVmac239 *nef*; and gray, disrupted *nef* gene. (**C, D**) Nef-mediated enhancement of viral spread in PBMCs. Percentages of virally infected GFP+ cells (M) and levels of p24 capsid antigen detected in the culture supernatants (N) of PBMCs infected with proviral constructs expressing *nef* alleles from the indicated groups of HIV-1-infected individuals or control *nef* alleles. Shown are average values (±SEM) for the entire group of HIV-1 *nef* alleles from viremic individuals with non-progressive (n = 16) or progressive (n = 10) infection. The results were confirmed in an independent experiment. (**E**) Expression of CD69 and levels of apoptosis in PBMCs infected with HIV-1 Nef/eGFP constructs and stimulated with CD3/CD28 beads (upper panels) or PHA (lower panels). Values represent the levels of CD69 relative to cells infected with a *nef*-defective HIV-1 NL4-3 construct. D, PBMC donor; Co, compiled data. VNPs, green circles; VPs, black diamonds.
(JPG)

Figure S2 **Fraction of cell associated HIV levels in absolute CD4+ T cell subsets.** CD4+ T_CM_ cells (left), T_EM_ (center), and T_SCM_ (right) from VNPs and PPs were sorted by flow cytometry and quantitaive real-time PCR was used to determine the HIV infection frequency in each subset. Infection frequency was determined by copies of *gag* DNA/100 infected cells. Frequency in absolute CD4+ T cells was calculated by multiplying the fraction of infected cells by the corresponding absolute number of CD4+ T cells. *p* values from Mann Whitney T test (VNPs vs PPs). Line reflects median. Circles, VNPs; Squares, PPs.
(PDF)

Table S1 **Full list of genes increased in VNPs compared to PPs by microarray, representing 476 annotated transcripts that were differentially expressed between subsets.**
(XLSX)

Table S2 **Full list of genes increased in PPs compared to VNPs by microarray, representing 293 annotated transcripts that were differentially expressed between subsets.**
(XLSX)

Table S3 **Patient characteristics of VNPs and viremic progressors from Italian cohort for** ***nef*** **analysis.**
(JPG)

Text S1 **Results and methods for** ***nef*** **functional characterization.**
(DOCX)
