# Supplementary figures and images for: Limited HIV Infection of Central Memory and Stem Cell Memory CD4+ T Cells Is Associated with Lack of Progression in Viremic Individuals
Source: PLoS Pathog. 2014 Aug 28;10(8):e1004345. doi: 10.1371/journal.ppat.1004345 (PMC4148445; doi:10.1371/journal.ppat.1004345)

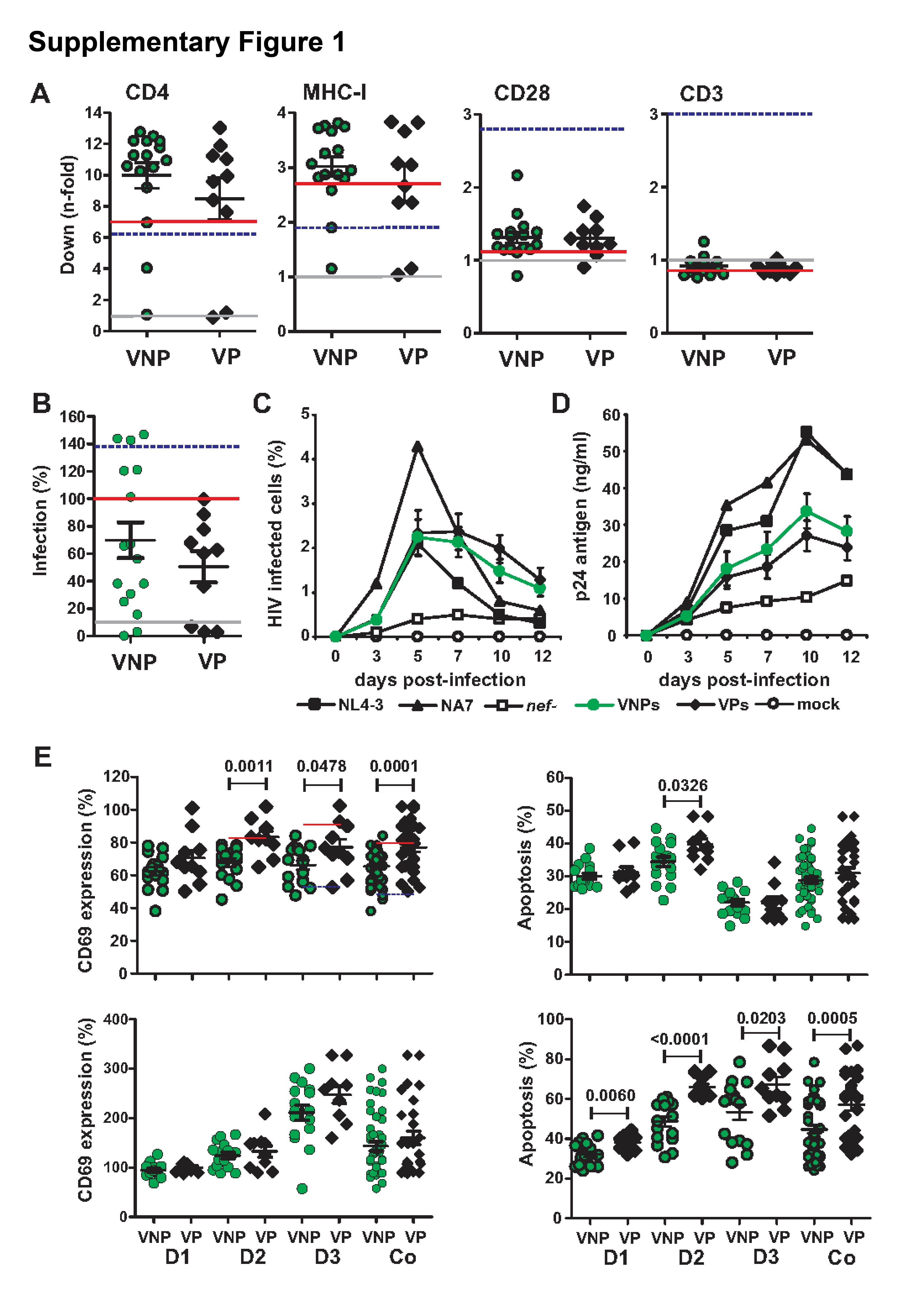

Supplement: Figure S1 — Functional characterization of nef alleles from VNPs and VPs. Nef functions were characterized in a separate cohort of VNPs and chronically HIV-infected viremic progressors (VPs). (A) Quantitation of Nef-mediated down-modulation of CD4, MHC-I, CD28 and CD3 on PBMCs infected with HIV-1 Nef/eGFP constructs. HIV-1 nef genes were grouped based on the patient characteristics and those from VNPs are color coded green. Each symbol indicates the n-fold down-modulation of the indicated receptor molecule by one of the 26 different NL4-3 proviral constructs. (B) Nef-mediated enhancement of virion infectivity. P4-CCR5 indicator cells were infected with proviral constructs expressing patient-derived nef alleles. Infections were performed with virus stocks containing 1 ng p24 antigen. Values represent the averages of two experiments compared to the infectivity of the virus expressing the NL4-3 Nef (100%). Values in panels A and B represent averages from two or three experiments, and the horizontal bars indicate average activities per group. The results obtained for the HIV-1 Nef/eGFP control constructs are indicated by lines: red, NL4-3 nef; broken blue, SIVmac239 nef; and gray, disrupted nef gene. (C, D) Nef-mediated enhancement of viral spread in PBMCs. Percentages of virally infected GFP+ cells (M) and levels of p24 capsid antigen detected in the culture supernatants (N) of PBMCs infected with proviral constructs expressing nef alleles from the indicated groups of HIV-1-infected individuals or control nef alleles. Shown are average values (±SEM) for the entire group of HIV-1 nef alleles from viremic individuals with non-progressive (n = 16) or progressive (n = 10) infection. The results were confirmed in an independent experiment. (E) Expression of CD69 and levels of apoptosis in PBMCs infected with HIV-1 Nef/eGFP constructs and stimulated with CD3/CD28 beads (upper panels) or PHA (lower panels). Values represent the levels of CD69 relative to cells infected with a nef-defecti [file ppat.1004345.s001.jpg]

# Cell Associated Virus (within Absolute CD4+ T cells)

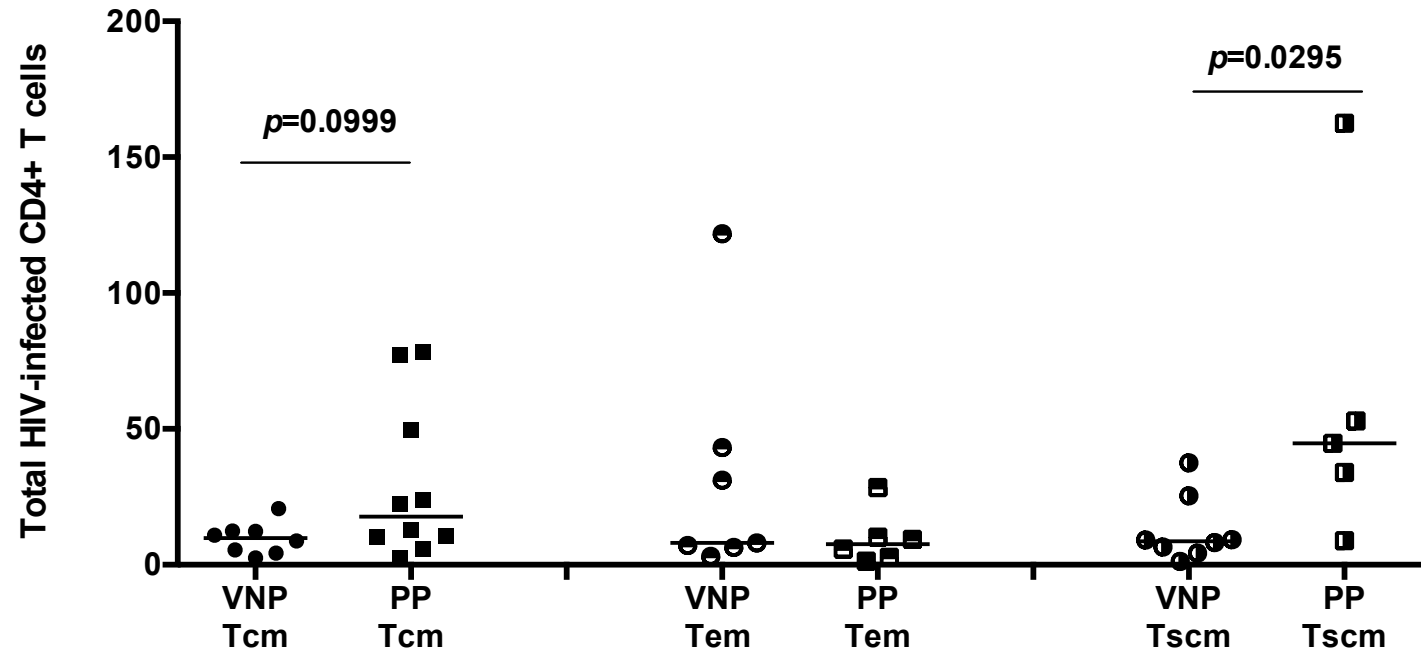

Supplement: Figure S2 — Fraction of cell associated HIV levels in absolute CD4+ T cell subsets. CD4+ TCM cells (left), TEM (center), and TSCM (right) from VNPs and PPs were sorted by flow cytometry and quantitaive real-time PCR was used to determine the HIV infection frequency in each subset. Infection frequency was determined by copies of gag DNA/100 infected cells. Frequency in absolute CD4+ T cells was calculated by multiplying the fraction of infected cells by the corresponding absolute number of CD4+ T cells. p values from Mann Whitney T test (VNPs vs PPs). Line reflects median. Circles, VNPs; Squares, PPs. (PDF) [file ppat.1004345.s002.pdf]

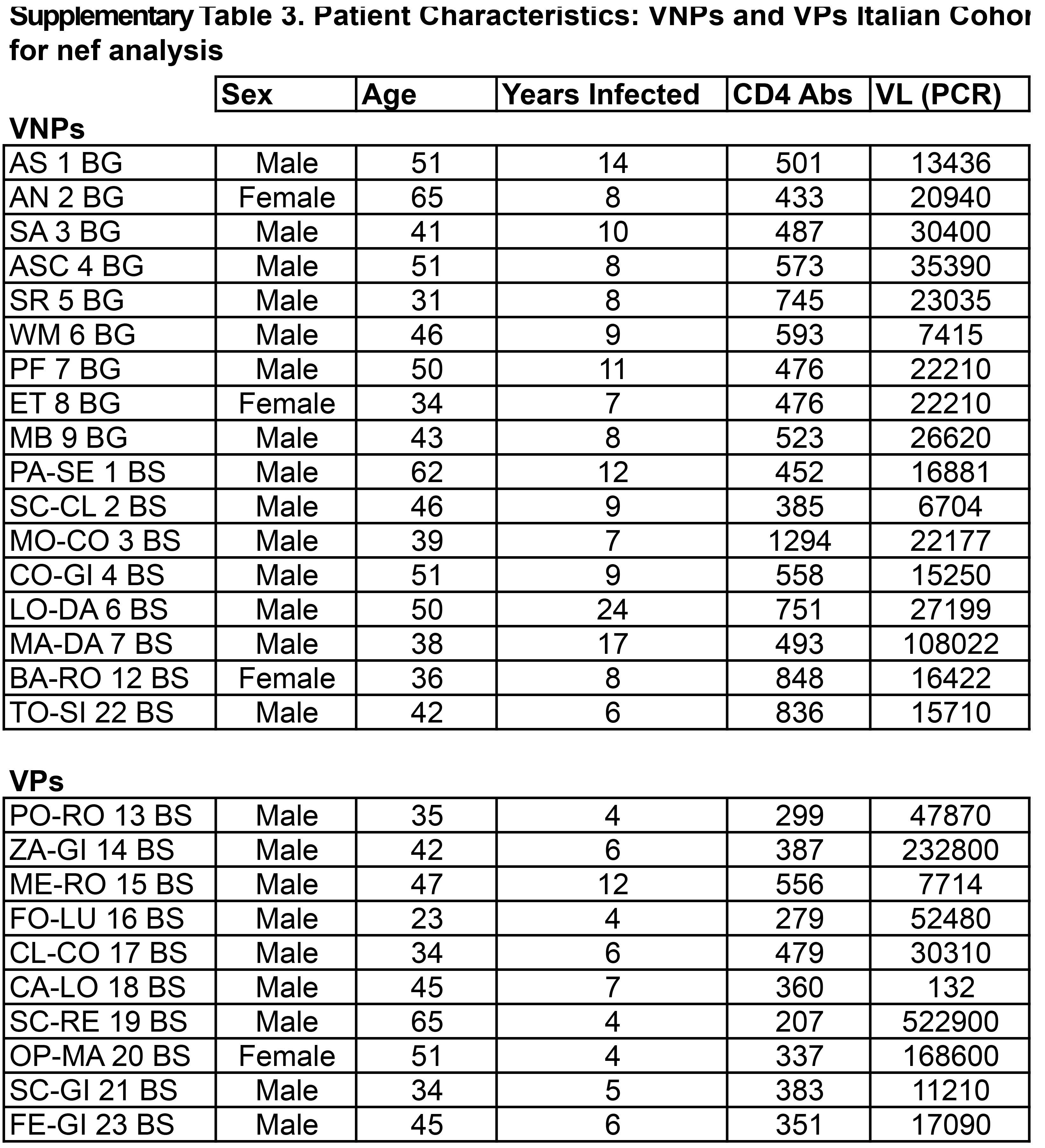

Supplement: Table S3 — Patient characteristics of VNPs and viremic progressors from Italian cohort for nef analysis. (JPG) [file ppat.1004345.s005.jpg]
