# Supplementary material for: Limited HIV Infection of Central Memory and Stem Cell Memory CD4+ T Cells Is Associated with Lack of Progression in Viremic Individuals
Source: PLoS Pathog. 2014 Aug 28;10(8):e1004345. doi: 10.1371/journal.ppat.1004345 (PMC4148445; doi:10.1371/journal.ppat.1004345)
Supplement: Text S1 — Results and methods for nef functional characterization. (DOCX) [file ppat.1004345.s006.docx]

**Figure S1 and S2 legends**

**Figure S1. Functional characterization of *nef* alleles from VNPs and VPs.** *Nef* functions were characterized in a separate cohort of VNPs and chronically HIV-infected viremic progressors (VPs). (**A**) Quantitation of Nef-mediated down-modulation of CD4, MHC-I, CD28 and CD3 on PBMCs infected with HIV-1 Nef/eGFP constructs. HIV-1 *nef* genes were grouped based on the patient characteristics and those from VNPs are color coded green. Each symbol indicates the *n*-fold down-modulation of the indicated receptor molecule by one of the 26 different NL4-3 proviral constructs. (**B**) Nef-mediated enhancement of virion infectivity. P4-CCR5 indicator cells were infected with proviral constructs expressing patient-derived *nef* alleles. Infections were performed with virus stocks containing 1 ng p24 antigen. Values represent the averages of two experiments compared to the infectivity of the virus expressing the NL4-3 Nef (100%). Values in panels A and B represent averages from two or three experiments, and the horizontal bars indicate average activities per group. The results obtained for the HIV-1 Nef/eGFP control constructs are indicated by lines: red, NL4-3 *nef*; broken blue, SIVmac239 *nef*; and gray, disrupted *nef* gene. (**C, D**) Nef-mediated enhancement of viral spread in PBMCs. Percentages of virally infected GFP+ cells (M) and levels of p24 capsid antigen detected in the culture supernatants (N) of PBMCs infected with proviral constructs expressing *nef* alleles from the indicated groups of HIV-1-infected individuals or control *nef* alleles. Shown are average values (±SEM) for the entire group of HIV-1 *nef* alleles from viremic individuals with non-progressive (n=16) or progressive (n = 10) infection. The results were confirmed in an independent experiment. (**E**) Expression of CD69 and levels of apoptosis in PBMCs infected with HIV-1 Nef/eGFP constructs and stimulated with CD3/CD28 beads (upper panels) or PHA (lower panels). Values represent the levels of CD69 relative to cells infected with a *nef*-defective HIV-1 NL4-3 construct. D, PBMC donor; Co, compiled data. VNPs, green circles; VPs, black diamonds.

**Figure S2. Fraction of cell associated HIV levels in absolute CD4+ T cell subsets.** CD4+ T_CM_ cells (left), T_EM_ (center), and T_SCM_ (right) from VNPs and PPs were sorted by flow cytometry and quantitaive real-time PCR was used to determine the HIV infection frequency in each subset. Infection frequency was determined by copies of *gag* DNA/100 infected cells. Frequency in absolute CD4+ T cells was calculated by multiplying the fraction of infected cells by the corresponding absolute number of CD4+ T cells. *p* values from Mann Whitney T test (VNPs vs PPs). Line reflects median. Circles, VNPs; Squares, PPs.

**Text S1**

**Nef alleles from VNPs are functionally active and do not efficiently suppress T cell activation and apoptosis**

The HIV-1 accessory Nef protein manipulates the activation and survival of virally infected T cells and plays an important role in AIDS pathogenesis (reviewed in [[1](#_ENREF_1),[2](#_ENREF_2)]). To investigate a possible association between Nef function and lack of disease progression in VNP, we amplified the nef genes from 16 VNP and 10 viremic progressors from an additional cohort (Supplemental Table 3). Since VNPs have decreased immune activation compared to chronically HIV-infected progressors [[3](#_ENREF_3),[4](#_ENREF_4)] but not when compared to PP with early infection, as shown above (Supplemental Figure 1), for this study we used a control cohort of progressively HIV-infected individuals who were infected on average 6.1 years (±2.56) (Supplemental Table 3). We cloned the 26 patient-representative *nef* genes into replication-competent HIV-1 NL4-3-based IRES-eGFP constructs, each co-expressing one Nef protein and eGFP from a bicistronic RNA. To determine the potency of *nef* alleles in modulating various receptors, we transduced human PBMCs with the proviral NL4-3-based IRES-eGFP constructs and analyzed them by flow cytometry. We found that *nef* alleles from both groups of HIV-1-infected individuals showed similar ability to down-modulate cell-surface expression of CD4 and MHC-I (Supplemental Figure 1A). Previous studies have shown that SIV_smm_ Nef-mediated down-modulation of CD3 and CD28 suppresses the activation of virally-infected T cells [[5](#_ENREF_5)] and may thus help to protect SIV_smm_-infected SMs against the loss of CD4+ T cells [[6](#_ENREF_6),[7](#_ENREF_7)]. However, none of the examined HIV-1 *nef* genotpes down-modulated TCR-CD3 and the effects on CD28 were generally very modest (Supplemental Figure 5A). Nef also facilitates viral spread directly by enhancing virion infectivity and by promoting viral replication in primary T cells (reviewed in [[1](#_ENREF_1),[2](#_ENREF_2)]). We found that most *nef* alleles from VNP and progressors enhanced virion infectivity, albeit with highly variable efficacy (Supplemental Figure 1B). We next examined the ability of *nef* alleles to promote viral spread in infected PBMC cultures. As reported previously [[5](#_ENREF_5)], the HIV-1 NL4-3 Nef/eGFP constructs are replication competent and have the advantage that the numbers of virally infected eGFP+ cells can readily be determined by flow cytometry. On average, *nef* alleles derived from VNP and VP were similarly efficient in promoting viral spread in PBMCs and in supporting p24 production by the virally infected cells (Supplemental Figure 1C, D).

Finally, Nef may modulate T cell activation and programmed cell death independently of CD3 or CD28 down-regulation via modulation of signal transduction pathways (reviewed in [[8](#_ENREF_8)]). To determine the effects of VNP and progressor Nefs on the responsiveness of primary human T cells we infected PHA-stimulated PBMCs with HIV-1 Nef-IRES-eGFP constructs and incubated them in the absence of exogenous stimuli until they expressed low levels of activation markers and eGFP. Thereafter, cells were treated with either anti-CD3/CD28 beads or PHA. We found that most HIV-1 *nef* alleles slightly reduced the induction of CD69 expression upon treatment with anti-CD3/CD28 beads (Supplemental Figure 1E, upper left). Interestingly, VNP Nefs were usually slightly more active in suppressing early T cell activation compared to those from progressively-infected individuals after stimulation with CD3/CD28 beads. In comparison, the effects of Nef in PHA-treated PBMC cultures were more variable, with *nef* alleles from both group of patients behaving similarly in terms of CD69 induction in PHA-treated PBMCs (Supplemental Figure 5E, lower left). More interestingly, expression of VNP Nefs was frequently associated with lower levels of apoptosis than VP Nefs (Supplemental Figure 1L), with this difference being more consistent after PHA treatment. Overall these results suggest that VNP *nef* alleles are slightly more effective in suppressing early T-cell activation and programmed cells death than VP *nef* alleles but much less active than SIVsmm Nefs.

**Methods within Text S1:**

**PCR amplification of *nef***

Viral RNA was extracted from plasma samples of 26 individuals (16 VNPs and 10 Ps) using the RNeasy minikit (Qiagen, Hilden, Germany) and cDNA was synthesized using the One-Step RT-PCR kit (Invitrogen). *Nef* genes were amplified in bulk using nested PCR with the outer primers: 5´-GTGGAACTTCTGGGACGCAGGGGGTGGG-3´ and 5´-GCAAAAAGCAGCTGCTTATATGCAGCATCTGAGG-3´ and the inner primers 5´-CAAAGAGCTTATAGAGCTATTCTCCACATACC-3´ and 5´-CCTGGAAAGTCCCC-AGCGGAAAGTCCCTTG-3´. The resulting PCR products were purified using the peqGold Cycle Pure kit (peqLab, Wilmington, DE) and sequenced directly using the inner PCR primers. Five single clones were sequenced and a single clone with the highest homology to the pool sequence was chosen for analysis. Thus, the nef sequences should be representative for the respective patient.

**Proviral constructs**

Generation of HIV-1 (NL4-3-based) proviral constructs carrying the patient-derived *nef* genes followed by an IRES element and the *eGFP* gene were generated by splice overlap extension PCR as described previously [[5](#_ENREF_5),[9](#_ENREF_9)]. All proviral HIV-1 constructs were verified by sequence analysis. Otherwise isogenic proviral constructs carrying the HIV-1 NL4-3 or NA7 *nef* alleles disrupted *nef* genes were used as controls and have been described previously [[5](#_ENREF_5),[9](#_ENREF_9)].

**Cell culture**

293T, P4-CCR5, TZM-bl and THP-1 were cultured as described elsewhere [[9](#_ENREF_9),[10](#_ENREF_10)]. Peripheral blood mononuclear cells (PBMCs) from healthy human donors were isolated using lymphocyte separation medium (Biocoll Separating Solution, Biochrom, Cambridge, UK). CD4^+^ T cells were isolated using the human CD4^+^ T cell RosetteSep kit (Stemcell Technologies) following the protocols provided by the manufacturers. The cells were stimulated for 3 days with PHA (2 µg/ml) and IL-2 (10 ng/ml) or with human CD3/CD28 T-Activator Dynabeads (Invitrogen) at a cell-to-bead ratio of 1:1 and IL-2 prior to transduction or infection.

**Virus stocks and transduction**

To generate viral stocks, 293T cells were co-transfected with the proviral HIV-1 constructs either alone (for infectivity or replication assays) or together with a plasmid (pHIT-G) expressing the Vesicular Stromatitis Virus G protein (VSV-G) [[5](#_ENREF_5)]. The latter was used to achieve comparably high initial infection levels for flow cytometric analysis. The medium was changed after overnight incubation, and virus was harvested 24 h later. For some assays (infectivity and replication) virus stocks were quantified using a p24 antigen capture assay provided by the NIH AIDS Research and Reference Reagent Program. All cell types were transduced with NL4-3-based proviral constructs coexpressing the various Nef proteins and eGFP as described previously [[5](#_ENREF_5)]. For the detection of CD4 down-modulation, proviral HIV-1 constructs defective in *VPu* and *env* (Wildum *et al*., 2006) were used, since both gene products also reduce CD4 cell surface expression and thus mask the effect of Nef. Flow cytometric analysis was performed at 3 days post-transduction.

***Nef* flow cytometric analysis**

CD3 (BD Pharmingen), CD28 (BD Pharmingen), and MHC-I (Dako, Carpinteria, CA) expression in PBMC and CD4 (Invitrogen) expression in purified CD4^+^ T cells transduced with HIV-1 (NL4-3) constructs coexpressing Nef and eGFP was measured as described previously [[5](#_ENREF_5)]. For quantification of Nef-mediated modulation of the specific surface molecules, the levels of receptor expression were determined for cells expressing eGFP. The extent of up- or down-modulation (*n*-fold) was calculated by dividing the mean fluorescence intensity (MFI) obtained for cells transduced with the *nef*-negative NL4-3 control viruses by the corresponding values obtained for cells transduced with viruses coexpressing Nef and eGFP.

**Infectivity assays**

Virus infectivity was determined using TZM-bl and P4-CCR5 cells as described [[10](#_ENREF_10)]. Briefly, the cells were plated in 96-well-dishes in a volume of 100 µl and infected after overnight incubation with virus stocks containing 1 ng of p24 antigen produced by transiently transfected 293T cells. Two days post-infection viral infectivity was detected using the Gal screen kit from TROPIX as recommended by the manufacturer. ß-galactosidase activities were quantified as relative light units per second (RLU/s) using the Orion Microplate Luminometer (Titertek Berthold, Pforzheim, Germany).

**Viral replication in PBMC**

To determine the ability of the patient-derived Nefs to enhance viral replication, 3 x 10^5^ pre-stimulated PBMC per well were sown out in 48-well dishes and infected with 293T cell derived virus stocks containing 4 ng of p24 antigen. Supernatants were harvested to monitor virus production by p24 ELISA and aliquots of the cells were obtained at 3, 5, 7, 10 and 12 days post-infection to determine the number of virally infected GFP+ cells by flow cytometric analysis.

**Cellular activation and apoptosis**

PBMC were first stimulated with CD3/CD28 T-activator Dynabeads at a cell-to-bead ratio of 1:1 and IL-2 for 3 days. Subsequently, the cells were transduced with the various HIV-1 eGFP/Nef constructs and cultured in RPMI1640 (10% FCS, 10 ng/ml IL-2) for another 3 days. At this time the PBMC expressed very low levels of CD69 and IL-2R and hence had a resting phenotype. Thereafter, the PBMC were treated a second time with PHA and CD69 (BD Pharmingen) expression levels were measured by FACS analysis one and three days later. The frequency of virally infected apoptotic cells was determined using the AnnexinV (AnV) Apoptosis Detection Kit (BD Bioscience) as recommended by the manufacturer.

**References within Text S1**

1. Kirchhoff F, Schindler M, Specht A, Arhel N, Munch J (2008) Role of Nef in primate lentiviral immunopathogenesis. Cell Mol Life Sci 65: 2621-2636.

2. Arien KK, Verhasselt B (2008) HIV Nef: role in pathogenesis and viral fitness. Curr HIV Res 6: 200-208.

3. Choudhary SK, Vrisekoop N, Jansen CA, Otto SA, Schuitemaker H, et al. (2007) Low immune activation despite high levels of pathogenic human immunodeficiency virus type 1 results in long-term asymptomatic disease. J Virol 81: 8838-8842.

4. Rotger M, Dalmau J, Rauch A, McLaren P, Bosinger SE, et al. (2011) Comparative transcriptomics of extreme phenotypes of human HIV-1 infection and SIV infection in sooty mangabey and rhesus macaque. J Clin Invest 121: 2391-2400.

5. Schindler M, Munch J, Kutsch O, Li H, Santiago ML, et al. (2006) Nef-mediated suppression of T cell activation was lost in a lentiviral lineage that gave rise to HIV-1. Cell 125: 1055-1067.

6. Schindler M, Schmokel J, Specht A, Li H, Munch J, et al. (2008) Inefficient Nef-mediated downmodulation of CD3 and MHC-I correlates with loss of CD4+T cells in natural SIV infection. PLoS Pathog 4: e1000107.

7. Khalid M, Yu H, Sauter D, Usmani SM, Schmokel J, et al. (2012) Efficient Nef-mediated downmodulation of TCR-CD3 and CD28 is associated with high CD4+ T cell counts in viremic HIV-2 infection. J Virol 86: 4906-4920.

8. Skowronski J, Greenberg ME, Lock M, Mariani R, Salghetti S, et al. (1999) HIV and SIV Nef modulate signal transduction and protein sorting in T cells. Cold Spring Harb Symp Quant Biol 64: 453-463.

9. Schindler M, Wurfl S, Benaroch P, Greenough TC, Daniels R, et al. (2003) Down-modulation of mature major histocompatibility complex class II and up-regulation of invariant chain cell surface expression are well-conserved functions of human and simian immunodeficiency virus nef alleles. J Virol 77: 10548-10556.

10. Munch J, Rajan D, Schindler M, Specht A, Rucker E, et al. (2007) Nef-mediated enhancement of virion infectivity and stimulation of viral replication are fundamental properties of primate lentiviruses. J Virol 81: 13852-13864.
